# Dynamic Response of Buried Pressurized Pipelines When Subjected to Transverse Impact Loading: Experimental Study

**DOI:** 10.3390/ma18061362

**Published:** 2025-03-19

**Authors:** Ruobing Wu, Kai Zhuang, Yuchao Yang, Zhijie Wu, Feng Liu

**Affiliations:** 1 Shandong Key Laboratory of Civil Engineering Disaster Prevention and Mitigation, Shandong University of Science and Technology, Qingdao 266590, China; 2 School of Civil Engineering, Southeast University, Nanjing 211189, China

**Keywords:** pressurized pipelines, burial environment, soil type, pipeline burial depth, deformation response

## Abstract

This study investigated the dynamic behavior of pressurized pipelines under impact loads in various burial environments. A total of 60 tests were conducted using a drop-weight impact testing apparatus, with high-speed cameras and sensors used to collect data throughout the experiments. This approach enabled a thorough investigation of the effects of the soil type, burial depth, and internal pressure on pipeline behavior. The results show that pipeline deformation decreases as burial depth and soil stiffness increase. Under similar conditions, unpressurized pipelines exhibit significantly greater plastic deformation than pressurized pipelines, indicating superior energy absorption. The application of internal pressure leads to a marked increase in the peak impact force compared to scenarios without internal pressure. Fluctuations in internal volume cause pressure variations, affecting the response of the pipeline. Additionally, higher soil stiffness results in increased peak impact force, particularly in loess environments, suggesting that stiffer soils improve pipeline impact resistance. It is important to note that deeper burial does not always lead to reduced impact forces. In stiff soil conditions, greater burial depth may even amplify the impact force. These effects are closely associated with stress concentration under impact loads, changes in soil support capacity, and the influence of internal pressure. The test data and analysis provided in this paper will contribute to optimizing pipeline design and protective strategies, thereby enhancing their safety and reliability.

## 1. Introduction

Buried pipelines are essential for modern energy transmission and municipal infrastructure, serving in the transportation of oil, natural gas, water resources, and urban drainage systems. Their safety and reliability are crucial to a nation’s economic vitality and social stability. However, throughout their service life, these pipelines are often exposed to a range of external impact loads, including earthquakes, construction machinery, traffic, and explosive forces. Such impacts can induce localized or extensive deformation, potentially causing catastrophic failures such as ruptures or leaks, which could lead to significant economic damage and environmental contamination. Therefore, conducting thorough research on the impact response of buried pipelines is critical not only for refining design methodologies but also for enhancing safety assessments and disaster mitigation strategies.

Considerable research has been conducted on the impact response of buried pipelines, primarily focusing on load types, soil–pipeline interaction, and failure mechanisms. Early studies mainly relied on theoretical analysis and simplified models to explore the mechanical behavior of pipelines under impact conditions. Notably, Norman et al. [1,2,3] developed a comprehensive dynamic response theory for pipelines subjected to low-speed impact loads, providing a fundamental theoretical basis for further investigations into pipeline impact responses.

Chen et al. [4] carried out extensive studies on the behavior of steel pipes with fixed ends under a lateral impact, performing over 130 impact tests to examine the dynamic inelastic behavior of steel pipes with varying diameters, wall thicknesses, and impact locations (midspan, quarter span, and near supports). Their research yielded extensive data on impact velocity, impact force, displacement-time histories, permanent deformation, and various failure modes. Building on this dataset, Shen et al. [5] proposed a theoretical quasi-static analysis method to predict the response and failure behavior of both pressurized and unpressurized pipelines subjected to lateral impacts. This method incorporated the maximum strain criterion, strain rate effects, and an empirical formula for estimating the plastic hinge length. The theoretical predictions were consistent with experimental results regarding the impact energy threshold and maximum permanent deformation, confirming the applicability of the maximum strain criterion and the empirical hinge length formula in theoretical analyses.

Yang et al. [6] discovered that the impact location plays a crucial role in determining the damage level to buried pipelines, with midspan impacts causing the most severe damage. O’Rourke et al. [7] examined the deformation characteristics of buried pipelines subjected to seismic waves, emphasizing that these deformations are primarily influenced by the direction of seismic wave propagation and the soil–pipeline interaction. In a related study, Baker et al. [8] investigated the local buckling and fracture behavior of pipelines under explosive shock, highlighting the significant role of shock wave intensity and material properties in determining the failure modes.

Mechanical impact loads have also been widely studied. Zhu et al. [9] employed a nonlinear pipe–soil coupling contact model to analyze the dynamic response of buried pipelines under landslide-induced impact loads. Their study explored the effects of the rockfall impact velocity, pipeline burial depth, diameter-to-thickness ratio, and soil type on axial compressive strain. The results indicated that ellipsoidal rockfalls generate considerably higher impact forces compared to spherical counterparts of the same volume, with larger rock masses further amplifying the impact intensity. Trautmann et al. [10] conducted experimental investigations into the effects of varying soil properties on pipeline deformation, emphasizing the influence of soil stiffness and damping characteristics on the pipeline’s impact response. Zhang et al. [11] performed a comprehensive analysis of the deformation, stress, and strain of buried steel pipes subjected to impacts from hazardous rocks using finite element simulations. Their findings revealed that the pipeline’s cross-section undergoes significant deformation during the impact process, transforming from elliptical to peach-shaped and eventually to crescent- or hourglass-shaped. The study also systematically analyzed the buckling behavior of pipelines under both lateral and longitudinal inclined impacts.

In recent years, Choudhury et al. [12] have focused on the effects of seismic landslides and other ground deformation modes on buried pipelines, as well as the impact of ground vibrations from pipeline ruptures on surrounding pipelines. Lee [13] studied the seismic performance of buried natural gas pipelines under earthquake loading, revealing that strain responses at the pipeline ends are highly influenced by soil and boundary conditions. Notably, under soft clay soil conditions, the strain at the pipeline ends can reach up to 70% of the allowable limit. Arabzadeh [14] investigated the impact of burial depth on the deformation of subsea pipelines, concluding that increased burial depth results in both higher maximum impact loads and greater indentation depths. Dou [15] explored the effects of internal pressure on a pipeline’s resistance to impacts, finding that higher internal pressure leads to increased maximum impact forces, reduced lateral displacements, and lower absorbed impact energy.

Despite there having been significant progress in studying the impact response of buried pipelines, such as Alsos H.S.’s [16] research on the effects of impacts on buried pipelines, which concluded that pipeline deformation under external impacts was primarily influenced by the mass and velocity of the impacting object rather than its kinetic energy, several limitations persist in current studies. First, existing research tends to focus on individual influencing factors and lacks multi-factor coupling analysis, particularly regarding the impact response of pipelines at varying burial depths. However, in practical engineering applications, pipelines are often subjected to multiple factors simultaneously. Second, current experimental methods face challenges in load application and data processing. For example, assessing the complex interactions between the soil layer and the pipeline during loading is difficult, and measuring changes in the internal pressure of pressurized pipelines presents additional challenges. These limitations hinder our understanding of pipeline impact response characteristics under real-world conditions. Therefore, further systematic research is urgently needed to uncover the multi-factor response mechanisms of buried pipelines under impact loads.

In this study, we aimed to investigate the deformation response mechanisms of buried pipelines under impact loads through experimental methods, focusing on key influencing factors such as the soil environment, burial depth, and internal pressure levels. The findings provide a scientific basis for safe design and disaster prevention in pipelines. The specific research objectives included: (1) conducting experimental studies to characterize the deformation of buried pipelines under various impact loads, thereby elucidating the role of pipeline–soil interaction in impact response; (2) analyzing the influence of soil properties, burial depth, and internal pressure levels on the impact response of pipelines based on experimental data; and (3) developing a theoretical model for the impact response of buried pipelines to support the safety assessment of pipelines based on the experimental results.

To achieve these objectives, for the present study, we designed and established an experimental setup, described in Section 2, to examine the impact response of buried pipelines, conducting impact tests with varying soil environments, burial depths, and internal pressure levels. Section 3 presents a comprehensive analysis of the data collected under typical operating conditions. Finally, the mechanisms of key influencing factors, including the soil environment, burial depth, and internal pressure levels, are discussed in conjunction with the experimental results.

## 2. Experiment Design and Apparatus

### 2.1. Test Specimens and Experimental Apparatus

This experiment employed thin-walled seamless circular pipes fabricated from API 5L X100 pipeline steel as test specimens, with a nominal wall thickness of 1 mm and a diameter-to-thickness ratio *D*/*t* = 25. The material conforms to the API 5L specifications for grade X100. Uniaxial tensile tests measured the yield stress, elastic modulus, and elongation at fracture of the experimental X100 steel batch as 763 MPa, 209 GPa, and 13.6%, respectively. The specimen dimensions include an overall length of 780 mm, span of 330 mm, and outer diameter of 25 mm. It should be emphasized that the findings of this study are strictly applicable to thin-walled pipe configurations.

**Figure 1:**
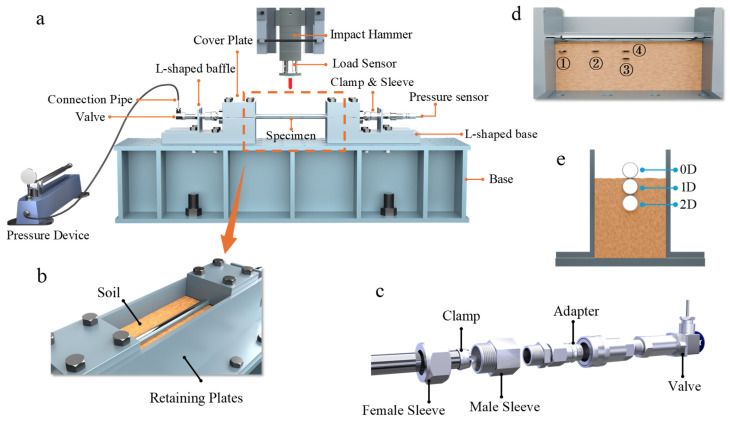
Experiment setup in present study. (**a**) Impact testing apparatus; (**b**) soil-retaining device; (**c**) connection method for pressurized pipeline; (**d**) layout of soil pressure sensors; (**e**) schematic representation of the burial depth for the test pipeline.

Impact loading was applied using a drop-weight testing apparatus (shown in Figure 1a), with the specimen positioned directly beneath the drop weight at mid-span. The specimen was secured in place by an L-shaped base and cover plate, which featured circular openings that matched the pipe’s outer diameter. The cover plate compressed the specimen against the L-shaped base using high-strength bolts, effectively restricting both radial and axial deformations during the test, thus establishing fixed boundary conditions at both ends of the pipe.

To improve the testing efficiency and eliminate residual welding stresses, pressure transmission and sealing were achieved using an adapter and clamps, as shown in Figure 1c. In this connection assembly, the male sleeve passes through the end of the pipe and locks the clamp by compressing the clamp and female sleeve together, applying radial pressure to secure the pipe’s end. The other end of the coupling is connected to a valve via an adapter, enabling the pressurization of the pipeline’s interior. At the opposite end of the pipe, the valve is replaced with a pressure transmitter, which monitors and records the internal pressure in real time.

To evaluate the interdependent influences of the soil type, burial depth, and internal pressure on pipeline impact behavior, a standardized experimental protocol was implemented with a fixed energy input across all test conditions. A 36.2 kg drop-weight apparatus with a 25 mm diameter hemispherical drop hammer served as the controlled energy source. The impact energy of 266 J was systematically achieved by releasing the mass from a calibrated 750 mm elevation, calculated through fundamental gravitational potential energy principles. This methodology maintained consistent kinetic energy transfer to test specimens while varying environmental parameters, enabling the direct comparison of soil–pipeline interaction mechanisms under equivalent mechanical loading conditions.

The present study focused on the dynamic response of pipelines in buried environments; thus, a specialized burial fixation device was designed (shown in Figure 1b). This apparatus consists of two soil-retaining steel plates, each 600 mm in length and 5 mm in thickness, arranged axially along the pipeline. The plates are securely attached to the base of the drop weight using bolts to ensure a sufficient soil burial environment for the test.

### 2.2. Buried Environments

To examine the response characteristics of pipelines buried in different soil types, typical soils such as soft clay, sand, and loess were selected for the present impact tests. Before conducting the impact tests, material tests were performed on the three selected soil types to determine their basic mechanical parameters, as detailed in Table 1. Additionally, a series of tests were conducted on suspended pipelines as a control for burial effects. To evaluate the coupling influence of soil and internal pressure, in addition to conducting tests on empty pipelines, a series of tests was also performed on pipelines pressurized to 10 MPa using the device shown in Figure 1a.

During the experiments, the burial depth of the pipeline was controlled by adjusting the soil fill depth. To investigate the influence of burial depth on the pipeline’s response, three burial configurations were tested, as shown in Figure 1e. For clarity and ease of comparison, these configurations are referred to as “0D”, “1D”, and “2D”, representing the pipeline located at the soil surface, buried at a one-diameter depth, and buried at a two-diameter depth, respectively.

### 2.3. Measurements and Test Procedures

During the experiments, the impact force and internal pressure variations were measured using the load cell, pressure sensors, and dynamic acquisition system shown in Figure 1a. Based on these measurements, digital image correlation (DIC) technology was used to track the digital speckle on the surface of the drop weight, allowing for precise displacement measurements. These data were synchronized with the timestamps from the data collected by the load sensor to derive relationships between impact loads, internal pressures, and vertical displacements covering the whole response of the pipeline.

To capture changes in soil pressure during the impact process, as shown in Figure 1d, four pressure sensors were embedded in the soil. These sensors were positioned at distances of 30 mm and 60 mm from the bottom of the pipeline. Specifically, Sensor ① was placed directly beneath the left side of the pipeline near its end, Sensor ② was positioned at a quarter span directly below the pipeline, and Sensors ③ and ④ were placed directly beneath the midpoint (impact location) of the pipeline.

The following is a brief description of the experimental procedure. The connecting sleeves at both ends of the pipeline specimens were attached to the corresponding connectors. For the pressurized pipeline, the quick connector on the pressure sensor side was released. Water was then injected into the pipeline through the pressurization apparatus until a stable flow emerged from the quick connector outlet, ensuring that the pipeline was completely filled with water and free from residual air. The pipeline specimens were fixed as described earlier, and the internal pressure reached the predetermined level. The soil was layered and compacted to the required depth in the burial area, with each layer being 30 mm thick. Simultaneously, the pressure sensors were buried at their designated depths.

After raising the drop weight to the specified height, all sensor readings were calibrated, and the drop weight was released to impact the pipeline specimen. A high-speed camera recorded the deformation process of both the pipeline and the surrounding soil. The internal pressure sensors monitored changes in the internal pipe pressure, while the soil pressure sensors collected data on variations in the soil layer pressure during the impact process. At the end of the experiment, before the specimen was removed from the self-restraint device, a vernier caliper was used to measure the vertical deflection, lateral width, and remaining thickness of the impacted area of the pipeline.

### 2.4. Loading Conditions

The experiments were organized into four groups, i.e., unburied, soft clay, sand, and loess groups. In the unburied group, two sets of tests were performed: one on an empty pipeline and another on a pipeline filled with water at 10 MPa. The remaining three groups underwent crossover testing for various burial depths and internal pressure conditions. Each configuration was repeated three times, and the average data were taken as the final results for that condition. This systematic experimental design facilitates our understanding of the mechanisms by which different factors influence the dynamic impact response of pipelines. All the loading conditions in the present study are summarized in Table 2.

## 3. Experimental Results and Analysis

### 3.1. Transient Response Process

A total of 60 low-speed impact tests were conducted across four groups. During these experiments, the pipeline exhibited varying degrees of permanent inelastic deformation without evident failure, a phenomenon also observed in studies by other researchers [17,18,19,20].

**Figure 2:**
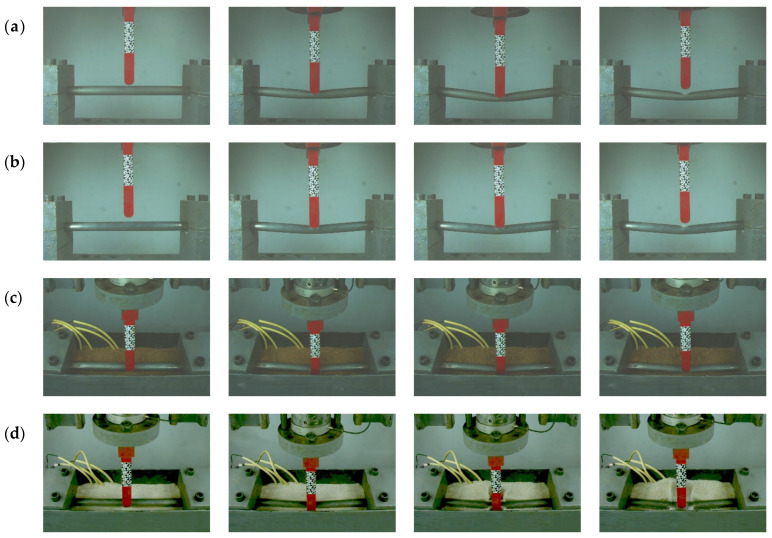

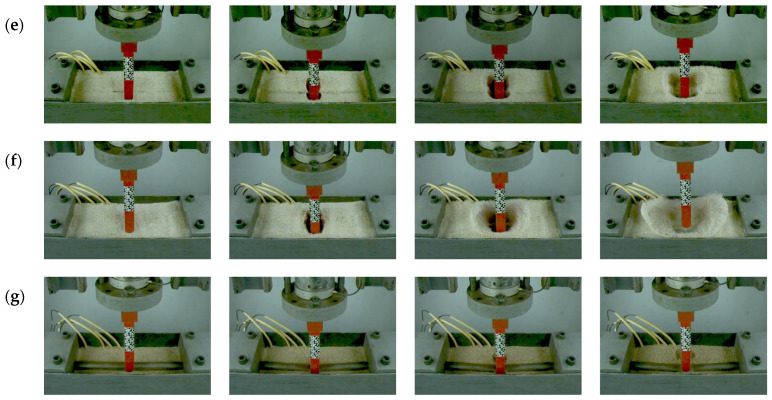
High-speed photographs of pipeline in typical loading conditions; (**a**) unburied, empty; (**b**) unburied, pressurized; (**c**) soft clay, empty, 0D; (**d**) sand, empty, 0D; (**e**) sand, empty, 1D; (**f**) sand, empty, 2D; (**g**) loess, empty, 0D.

Figure 2 captures the deformation process of the pipeline’s impact response under typical conditions, with high-speed camera images taken at 15 ms intervals. Comparing Figure 2a and 2b, the deformation characteristics of empty and pressurized pipelines in an unburied condition are evident. It can be observed that under impact loads, both conditions underwent two stages: an initial stage dominated by local deformation followed by a stage characterized by global deformation. Under the same impact conditions, the deformation response of the empty pipeline was significantly greater than that of the pressurized pipeline, indicating that the internal pressure plays a significant role in resisting pipeline deformation. These experimental observations align with findings from Norman [3], Dou [15], and Özcebe [17].

In the sand environment, Figure 2d–f show the impact processes of empty pipelines buried at depths of 0D, 1D, and 2D, as recorded by high-speed cameras. The results indicate that soil particles surrounding the pipeline experienced splashing phenomena due to the impact, which became more pronounced with greater burial depths. As shown in Figure 2c,d,g, under the same initial impact kinetic energy conditions, pipelines buried in sand exhibited larger deformation responses and significant soil splashing, suggesting weaker buffering effects compared to those in soft clay and loess. In contrast, pipelines embedded in soft clay showed significantly reduced deformation under the same conditions, indicating the stronger protective capacity of soft clay. Furthermore, pipelines buried in loess exhibited the smallest deformation response, with less pronounced soil splashing compared to sand. These results highlight the significant impact of soil type on the impact resistance performance of pipelines.

### 3.2. Pipeline Deformations

**Figure 3:**
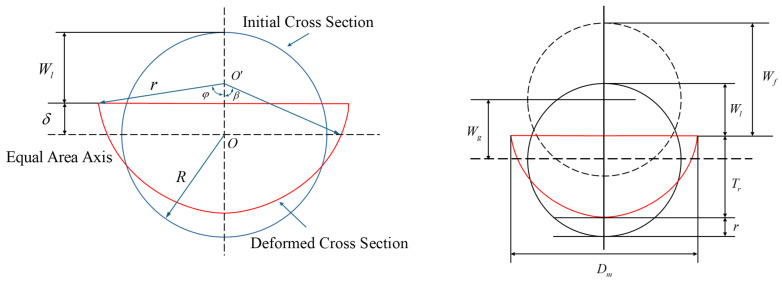
Schematic diagram of global and local deformation and key parameters [1].

**Figure 4:**
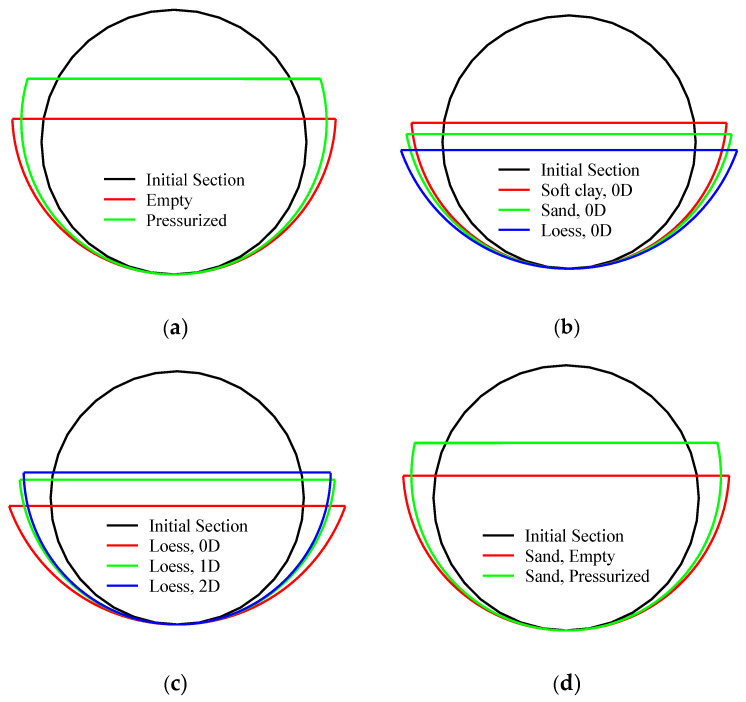
Comparison of the configuration of cross-sections in different loading conditions. (**a**) Unburied empty pipelines and pressurized pipelines; (**b**) pipelines in different soil environments; (**c**) pipelines with different burial depths; (**d**) pipelines with and without internal pressure before the impact test.

As shown in Figure 3, referring to the global and local deformation theory proposed by Norman et al. [1], the deformation characteristics of the pipeline under external impact loading are represented by the total displacement $W_{f}$, which can be further decomposed into the local deformation $W_{l}$ and global deformation $W_{g}$. According to Equations (1)–(6), the local deformation $W_{l}$ can be calculated by measuring the transverse width $D_{m}$ and vertical residual height $T_{r}$ of the deformed cross-section. The smaller the ratio of $W_{l}/W_{g}$, the greater the proportion of global deformation in the pipeline’s final state. Conversely, a larger ratio suggests that the pipeline’s final configuration is dominated by local deformation. $W_{l}$ and $W_{g}$, as well as their ratio (i.e., $W_{l}/W_{g}$) under each experimental condition, are summarized in Table 3, and the deformed configuration of the pipeline’s cross-section is demonstrated in Figure 4. From the variation trend in these test data, we can identify the response rules of such pipelines and recognize their underlying mechanisms.

(1) $$r=T_{r}\left\{1+{\left(D_{m}/2T_{r}\right)}^{2}\right\}/2$$

(2) β=πR/2r

(3) $$\cos\phi =1-T_{r}/r$$

(4) $$\delta =r\left(\cos\beta -\cos\phi \right)$$

(5) $$W_{l}=R-\delta$$

(6) $$W_{g}=W_{f}-W_{l}$$

The test results summarized in Table 3 and illustrated in Figure 4b indicate that as the soil hardness increases, the ratio of $W_{l}/W_{g}$ generally increases, suggesting that the pipeline’s deformation transitions from being predominantly influenced by global deformation in soft clay to being primarily governed by local deformation in a loess environment. This trend may be attributed to the fact that harder soils more effectively transmit energy to the pipeline during impact. This efficient energy transfer increases the pressure near the point of force application on the pipeline, leading to deformation in localized areas. Additionally, hard soils exert stronger constraints on the pipeline, limiting its global deformation; therefore, the constraint exerted by the soil on the pipeline increases under external forces, causing significant local deformation in areas with concentrated stress, while the global deformation remains relatively small.

Further, the burial depth of the pipeline also significantly affects the final deformation morphology. As demonstrated in Figure 4c, when the burial depth increases, the ratio of $W_{l}/W_{g}$ generally increases, indicating a shift from predominantly local deformation to global deformation. This may be attributed to the following factors: (1) Greater burial depth results in additional pressure from the overlying soil on the pipeline. This pressure enhances the constraining effect on the pipeline and concentrates stress in localized regions, leading to local deformation. (2) With deeper burial, the kinetic energy from the impact gradually dissipates after passing through thicker layers of soil. As a result, although the impact force remains, the effective energy applied to the pipeline is reduced, causing localized deformation in critical areas, while the global deformation remains minimal. (3) Deeper burial strengthens the surrounding soil’s ability to constrain the pipeline. The solid soil restricts the pipeline’s free deformation, and when external impacts occur, the pipeline tends to experience local buckling or compression near the point of force application, while other areas maintain stability.

Finally, the presence of internal pressure can significantly reduce the ratio of $W_{l}/W_{g}$, indicating that the global deformation mode will dominate for pressurized pipelines. This phenomenon is consistent with the conclusions drawn by other researchers (e.g., Norman et al. [1,3] and Shen et al. [19]). From observing Figure 4d, we can deduce that several factors may contribute to this effect. (1) The internal pressure is uniformly distributed within the pipeline and can counteract some of the externally applied pressure. When the pipeline experiences an external impact, the internal pressure provides additional resistance, helping the pipeline maintain stability and reducing deformation in localized areas. (2) The stress state of a pressurized pipeline changes when subjected to forces. Internal pressure alters the stress state of the pipeline walls, including hoop and axial stresses, thereby enhancing the pipeline’s ability to resist externally applied forces. This uniform distribution of stress helps mitigate local deformation. (3) The internal pressure increases the overall rigidity of the pipeline, causing its deformation mode under external loads to shift toward whole bending rather than localized plastic deformation and then helps disperse the effects of external forces on the whole pipeline. (4) The internal pressure alters the dynamic characteristics of the pipeline, enhancing its impact resistance. The global deformation mode enables the pipeline to better absorb and dissipate external energy, thereby reducing the potential damage caused by concentrated stresses.

### 3.3. Energy Dissipation in Pipelines

**Figure 5:**
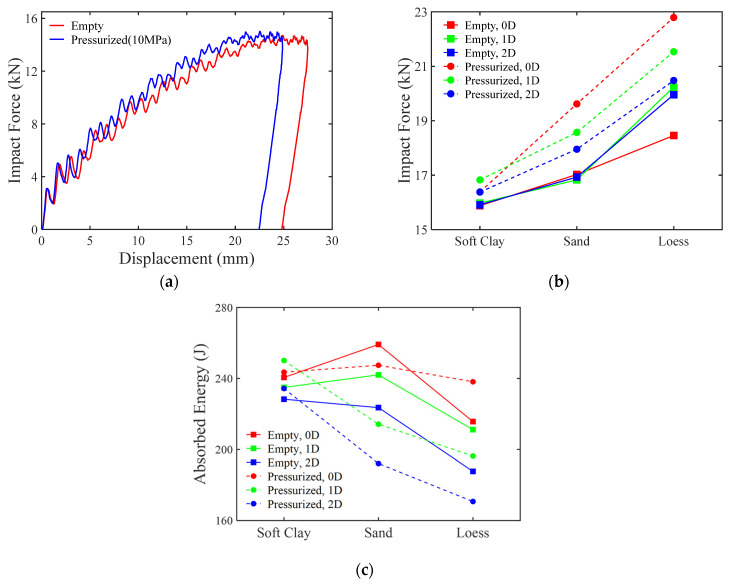
The variation in impact force and absorbed energy in different conditions. (**a**) Load–displacement relationship for the pipeline under no soil conditions; (**b**) maximum impact force for different soil environments; (**c**) comparison of energy absorption in pipelines under different conditions.

The contact force and deflection at the impact point on the pipeline are key variables for evaluating the response of the pipeline. As shown in Figure 5a, when an empty pipeline is struck by a rigid dropping hammer, both the impact force and deflection displacement gradually increase as the hammer descends. These values reach their maximum once the kinetic energy has been fully dissipated, after which the pipeline undergoes elastic rebound and exhibits permanent plastic deformation.

The area enclosed by the load–displacement curve of the free-falling impact body holds significant physical meaning, as it represents the work carried out by the external force after the impact body contacts the specimen. In this experiment, the energy imparted by the falling hammer can be divided into two components: one part is the energy consumed due to the splashing of soil layers when the hammer contacts the soil; the other part is the energy absorbed between the pipeline and the soil after the hammer contacts the pipeline. If the starting point of the load–displacement curve is defined as the moment of contact between the impact body and the pipeline, then the area enclosed by this curve reflects the pipeline’s ability to absorb the impact energy.

As shown in Figure 5b, the presence or absence of internal pressure, types of soil, and burial depth significantly affect the impact response of pipelines. Specifically, the presence of internal pressure results in peak impact forces that are higher than those observed without internal pressure. The internal pressure within the pipeline forces it to release a portion of the energy along the unloading path after loading results in permanent plastic deformation. This manifests macroscopically as a relative reduction in the vertical deflection of the pipeline. However, for the impact body, the initial energy remains unchanged, which inevitably increases the impact force exerted by the impact body, further leading to a significant reduction in the pressure of the soil layer beneath the pipeline.

An increase in internal pressure typically indicates a reduction in the internal volume of the pipeline. When impacted at the midpoint, the local cross-sectional area of the pipeline decreases. Simultaneously, the presence of hydraulic forces generates circumferential tensile stresses at the ends of the pipeline, which increases the cross-sectional area at those locations. However, the external constraint imposed by the surrounding harder soil layers limits such deformation. As a result, this leads to an increase in the internal pressure of the pipeline, as discussed in Section 3.4.

Conversely, an increase in burial depth does not necessarily lead to a reduction in impact force. Although experimental data from pressurized pipelines show a negative correlation between burial depth and peak impact force, no significant negative correlation exists for empty pipelines. In hard soil environments, increasing the burial depth can actually result in an increase in impact force. This may be attributed to the enhanced load transfer performance of the overlying soil due to increased soil hardness.

The test results shown in Figure 5c indicate that the soil environment significantly affects the pipeline’s energy absorption. The energy absorption of the pipeline under the 0D condition is substantially higher than that under other burial conditions, with a clear decreasing trend in energy absorption as the burial depth increases. This suggests that the overlying soil layer effectively reduces the external impact energy. Notably, changes in the soil environment have a considerable influence on the pipeline’s energy absorption capacity. Specifically, as the soil hardness increases, the average energy absorption decreases from 239 J in soft clay to 217 J in loess. However, when the density of the soil decreases, as in the case of the least dense sand, almost all of the falling hammer’s energy (266 J) is absorbed by the pipeline (262 J) under the 0D condition, indicating that the underlying soil layer provides minimal support to the pipeline. Nonetheless, as the burial depth in sandy soil increases, the energy absorption of the pipeline significantly declines, although sand still exhibits good cushioning ability.

### 3.4. The Variation in Pressure Within the Pipeline and in the Surrounding Soil

**Figure 6:**
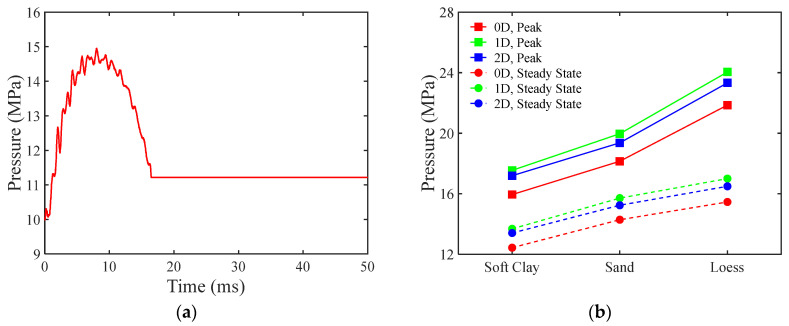
The evolution of internal pressure. (**a**) Time history of internal pressure in a pressurized pipeline in an unburied environment; (**b**) the value for peak and residual internal pressure for buried pipelines.

In contrast to the experiments conducted in the present study using water as the medium, Shen et al. [11] conducted impact tests on nitrogen-pressurized pipelines. As shown in Figure 6a, the initial internal pressure of an unburied pipeline increased from 10 MPa to 14.9 MPa before subsequently decreasing to 11.2 MPa during the whole response. In contrast to gas-filled pipelines, the dynamic response of liquid-filled pipelines is highly sensitive to changes in internal volume. For weakly compressible liquids, even minor volume changes can cause substantial pressure fluctuations, which, in turn, affect the magnitude of the hoop stress within the pipeline.

Comparing the cases of 0D and 1D burial depths, as shown in Figure 6b, both the peak and final internal pressure of the pipeline increase with greater burial depth. However, when the burial depth is further increased to 2D, both the peak and final internal pressures decrease. This may be due to the dissipation of some initial kinetic energy from the falling hammer before contact with the pipeline at greater burial depths, thus reducing the effective energy during the impact. Therefore, an optimal burial depth appears to enhance the dynamic response of buried pipelines under external impacts.

**Figure 7:**
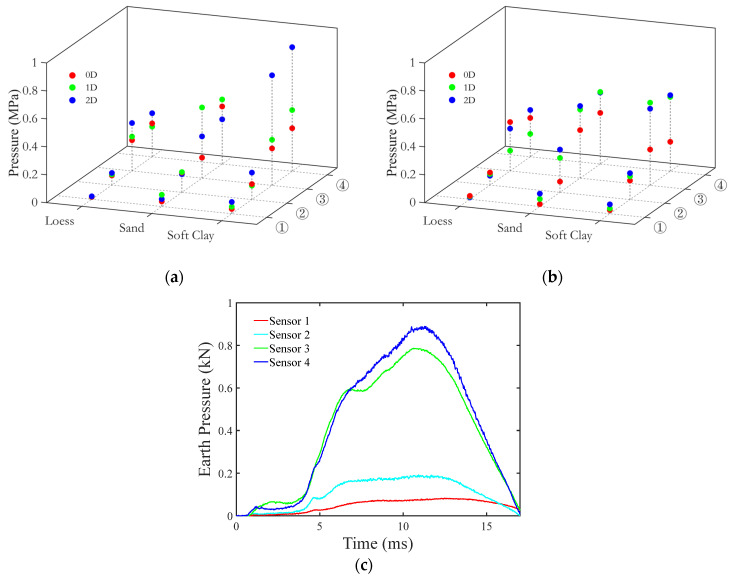
The evolution of soil pressure. (**a**) Test data for an empty pipeline; (**b**) test data for a pressurized pipeline; (**c**) time history of soil pressure for buried pressurized pipelines.

Figure 7 summarizes the variation in soil pressure in different loading conditions. As shown in Figure 7c, sensor ④, located closest to the midpoint of the pipeline, recorded a higher peak soil pressure than sensors ① and ②. This suggests that energy transmission from the impact point concentrates and diffuses outward, causing the soil to move closer to the impact point to experience a stronger impact response.

Furthermore, as shown in Figure 7a,b, the influence of different soil types on the soil pressure is evident. Hard soils are more effective at resisting pipeline deformation, thereby enhancing the soil’s energy response. As soil hardness increases, the values recorded by sensors ③ and ④ become increasingly similar, which can be attributed to the growing capacity of the soil to resist pipeline deformation. Consequently, the area of soil experiencing pressure also increases, and the enhanced soil hardness improves load transfer efficiency within the soil.

## 4. Conclusions and Outlook

In this study, we conducted experimental research to analyze the influence of the internal pressure, soil type, and burial depth on the impact response of pipelines. The following main conclusions can be drawn:

(1) The peak impact force of a pipeline with internal pressure is significantly higher than that under conditions without internal pressure. Liquid-filled pipelines are highly sensitive to changes in internal volume; even slight fluctuations can lead to substantial pressure variations, altering the whole response characteristics of the pipeline due to changes in the distribution of circumferential stress.

(2) As soil hardness increases, the peak impact force on the pipeline also rises, particularly in loess environments, where changes in the internal pressure of pressurized pipelines are especially pronounced. This indicates that higher soil hardness enhances the constraining capacity during the deformation process of the pipeline, resulting in elevated levels of internal pressure.

(3) An increase in burial depth does not always lead to a reduction in impact force. In hard soil environments, increasing the burial depth may enhance the load transfer performance of the overlying soil, thereby increasing the impact force. For empty pipelines, there is no evident negative correlation between the burial depth and peak impact force; however, an appropriate burial depth can optimize the dynamic response of pipelines under external impacts.

Future work could adopt a combined approach, with numerical simulation and experiments, to systematically analyze the roles of various variables. This will contribute to a better understanding of pipeline responses under complex loading conditions.

## Figures and Tables

**Table 1:** Mechanical properties of soils.

Parameter	Soft Clay	Sand	Loess
Standard Penetration Test N-value	13	7	17
Elastic Modulus	15 MPa	68 MPa	82 MPa

**Table 2:** Experimental conditions in the present study.

Serial Number	Soil Type	Pipeline Status	Depth
1	Unburied	Empty	-
2	Unburied	Pressurized	-
3	Soft Clay	Empty	0D
4	Soft Clay	Empty	1D
5	Soft Clay	Empty	2D
6	Sand	Empty	0D
7	Sand	Empty	1D
8	Sand	Empty	2D
9	Loess	Empty	0D
10	Loess	Empty	1D
11	Loess	Empty	2D
12	Soft Clay	Pressurized	0D
13	Soft Clay	Pressurized	1D
14	Soft Clay	Pressurized	2D
15	Sand	Pressurized	0D
16	Sand	Pressurized	1D
17	Sand	Pressurized	2D
18	Loess	Pressurized	0D
19	Loess	Pressurized	1D
20	Loess	Pressurized	2D

**Table 3:** Summary of global and local displacements for typical pipelines.

Experimental Conditions			$W_{f}$ (mm)	$D_{m}$ (mm)	$T_{r}$ (mm)	$W_{g}$ (mm)	$W_{l}$ (mm)	$W_{l}/W_{g}$
Soil	Status	Depth						
Unburied	Empty	-	24.8	30.5	14.7	16.01	8.79	0.549
Unburied	Pressurized	-	22.5	27.6	18.5	17.06	5.44	0.318
Soft Clay	Empty	0D	22	31.1	14.4	13.08	8.92	0.681
Soft Clay	Empty	1D	21.4	30.3	14.6	12.47	8.93	0.716
Soft Clay	Empty	2D	20.8	30.1	14.8	12.01	8.79	0.731
Sand	Empty	0D	19.7	32.1	13.3	10.05	9.65	0.960
Sand	Empty	1D	17.9	30.7	14.6	9.07	8.83	0.973
Sand	Empty	2D	16.6	30.5	14.8	7.92	8.68	1.095
Loess	Empty	0D	17.6	33.2	11.7	6.95	10.65	1.532
Loess	Empty	1D	15.0	31.1	14.3	5.99	9.01	1.504
Loess	Empty	2D	13.5	30.3	15.0	4.96	8.54	1.721
Soft Clay	Pressurized	0D	20.1	27.8	18.6	14.81	5.29	0.357
Soft Clay	Pressurized	1D	19.5	27.5	17.8	13.26	6.24	0.470
Soft Clay	Pressurized	2D	18.7	27.7	17.7	12.39	6.31	0.509
Sand	Pressurized	0D	16.8	28.0	18.6	11.55	5.25	0.454
Sand	Pressurized	1D	15.9	28.5	17.7	9.76	6.14	0.629
Sand	Pressurized	2D	14.7	28.1	14.5	5.07	9.63	1.899
Loess	Pressurized	0D	13.5	28.0	18.7	8.36	5.14	0.614
Loess	Pressurized	1D	12.2	28.8	17.6	6.01	6.19	1.029
Loess	Pressurized	2D	11.2	28.4	17.9	5.26	5.94	1.129

## Data Availability

The original contributions presented in the study are included in the article, further inquiries can be directed to the corresponding author.
